# Biomarkers for Chronic Kidney Disease Associated with High Salt Intake

**DOI:** 10.3390/ijms18102080

**Published:** 2017-09-30

**Authors:** Keiko Hosohata

**Affiliations:** Education and Research Center for Clinical Pharmacy, Osaka University of Pharmaceutical Sciences, Osaka 569-1094, Japan; hosohata@gly.oups.ac.jp; Tel.: +81-72-690-1271

**Keywords:** high-salt diet, hypertensive, normotensive, renal tubular injury chronic kidney disease, early biomarker, spontaneously hypertensive rats, Wistar Kyoto rats

## Abstract

High salt intake has been related to the development to chronic kidney disease (CKD) as well as hypertension. In its early stages, symptoms of CKD are usually not apparent, especially those that are induced in a “silent” manner in normotensive individuals, thereby providing a need for some kind of urinary biomarker to detect injury at an early stage. Because traditional renal biomarkers such as serum creatinine are insensitive, it is difficult to detect kidney injury induced by a high-salt diet, especially in normotensive individuals. Recently, several new biomarkers for damage of renal tubular epithelia such as neutrophil gelatinase-associated lipocalin (NGAL) and kidney injury molecule-1 (Kim-1) have been identified. Previously, we found a novel renal biomarker, urinary vanin-1, in several animal models with renal tubular injury. However, there are few studies about early biomarkers of the progression to CKD associated with a high-salt diet. This review presents some new insights about these novel biomarkers for CKD in normotensives and hypertensives under a high salt intake. Interestingly, our recent reports using spontaneously hypertensive rats (SHR) and normotensive Wistar Kyoto rats (WKY) fed a high-salt diet revealed that urinary vanin-1 and NGAL are earlier biomarkers of renal tubular damage in SHR and WKY, whereas urinary Kim-1 is only useful as a biomarker of salt-induced renal injury in SHR. Clinical studies will be needed to clarify these findings.

## 1. Introduction

Chronic kidney disease (CKD) is one of the serious health problems affecting millions of people and draining scarce health care resources. According to a systematic analysis, the total number of adults with CKD was approximately 220 million men and 270 million women [1]. Stratified across high-income, middle-income, and low-income countries, the majority of the CKD population is found in low- and middle-income countries; the population consists of only 48 million men and 62 million women in high-income countries, whereas it consists of about 170 million men and 210 million women in low- and middle-income countries [1]. This is partly because the majority of the world’s population lives in low- and middle-income countries. Recently, it has been reported that there was a welcome reduction in the prevalence of CKD in 2007–2012 compared with that in 1988–1994; however, the death rate remained largely stable in the general population and tended to decline among individuals with diabetes over the same time period [2]. Inappropriate lifestyle habits, especially dietary habits such as excessive salt intake, accelerate CKD via blood pressure (BP)-dependent and -independent mechanisms.

Sodium is an essential nutrient and one of the important cations in the extracellular fluid. It is vital in several physiologic processes such as the maintenance of extracellular volume and osmolality, membrane potentials, and several transmembrane transport processes. Several studies have suggested that salt intake deteriorates a pathophysiological renal function. In fact, a recently published double-blind controlled randomized trial in patients with CKD (stages 3 and 4) showed that dietary sodium restriction significantly decreased ambulatory BP by 10/4 mmHg, and consistent reductions in proteinuria and albuminuria were shown [3].

The importance of salt restriction is well recognized, and the World Health Organization (WHO) recommended that salt intake should be kept below 5 g/day in 2016, which is based on the WHO Guideline (WHO Guideline: Sodium intake for adults and children, 2012) in order to avoid health problems, especially those related to CKD as well as hypertension and the cardiovascular system. On the basis of the evidence showing associations of high dietary sodium intake with poor health outcomes, guidelines from the National Kidney Foundation Kidney Disease Outcomes Quality Initiative (NKF/KDOQI) state that there is strong evidence to support the recommendation of lowering salt intake to 90 mmol (less than 2 g) per day of sodium (corresponding to 5 g of sodium chloride) in adults, if not contraindicated [4]. However, average salt intake still exceeds 8 g per day in Japan, and it has been reported that Japanese hypertensive patients are poorly compliant with long-term salt restriction [5]. The failure to limit salt intake may lead to decreased renal function.

In this review, we refer to the salt-induced CKD under hypertension and normotension, from the classical to recent insights.

## 2. Association of High-Salt Diet with Blood Pressure and Chronic Kidney Disease in Hypertensive Patients and Animals

### 2.1. Association of High-Salt Diet with Blood Pressure in Hypertensive Patients

Classically, patients with essential hypertension show varied BP responses to dietary sodium intake; these responses may be salt-sensitive or salt-resistant, depending on the basis of absolute changes in BP [6]. In salt-sensitive hypertension, a high salt intake remarkably elevates BP [7]. This is confirmed by several clinical reports indicating that a reduction in salt intake causes a significant fall in BP in hypertensive patients [8,9,10]. In salt-sensitive hypertension, the accumulation of sodium in tissue has been presumed [11] to be accompanied by a commensurate retention of water to maintain the isotonicity of body fluids. Interestingly, skin also plays an important role as tissue in sodium storage; a high-salt diet in rats leads to interstitial hypertonic sodium accumulation in skin, resulting in increased density and hyperplasia of the lymphcapillary network. The mechanisms underlying these effects on lymphatics involve the activation of tonicity-responsive enhancer binding protein (TonEBP) in mononuclear phagocyte system cells infiltrating the interstitium of the skin [12].

On the other hand, in salt-resistant hypertensive patients, BP increases little during salt-loading due to large increases in external salt balance, and they continue to retain the excess sodium throughout the period of salt-loading [13].

In addition, several studies have demonstrated that high salt intake in obesity also plays a crucial role in the development of hypertension. In particular, childhood obesity is a serious problem, and it has been recently reported that childhood obesity is more prevalent in males than females, and severe obesity is associated with elevated BP [14]. Interestingly, there are sex-related differences in young adults aged 18 to 39 years rather than those aged ≥40 years; young adult men have substantially higher prevalence of pre-hypertension and lower awareness, treatment, and control compared with young adult women, and these sex-related differences were diminished in those ≥40 years of age [14].

### 2.2. Association of High-Salt Diet with the Kidney in Hypertensive Patients

BP elevation increases the risk of cardiovascular and cerebrovascular morbidity and mortality [15,16]. In addition to its effects on BP, salt intake per se accelerates target organ damages, leading to cardiovascular [17,18] and cerebrovascular [19] diseases. Renal damage is also induced by a high-salt diet, independent of BP. Clinical reports showed that a high salt intake accelerates albuminuria [18] and a decline in glomerular filtration rate [20] in hypertensive patients. Salt-sensitive hypertensive patients, such as those of older age [21], metabolic syndrome [22], African ancestry [23], and male patients [24] showed that greater dietary salt intake caused an increase in 24-h urine protein excretion [25]. On the other hand, in salt-resistant hypertensive patients, there was no significant change by salt-loading in 24-h urine protein excretion [25].

Reduced glomerular filtration rate and increased albuminuria are associated with poorer CKD prognosis [26]. In early stages, symptoms of CKD are usually not apparent. If diagnosed early (stage 1 to 3), the progression of CKD can be stemmed or delayed. However, no biomarkers have been reported for CKD in salt-sensitive hypertensive patients.

### 2.3. Association of High-Salt Diet with Blood Pressure and the Kidney in an Experimental Hypertensive Model

It is well accepted that excess salt exerts hypertension and promotes renal damage in spontaneously hypertensive rats (SHR), which is used worldwide as a characterized experimental model of naturally occurring hypertension [27]. Specifically, SHR are known to have a narrow afferent arteriolar lumen and to develop increased BP. Interestingly, SHR also have a reduced nephron number compared with their counterpart control, Wistar Kyoto rats (WKY). In SHR, dietary salt causes effects on hemodynamic factors characterized as renal hemodynamic and glomerular dynamic dysfunction [28]. Concurrently, excess dietary salt exerts additional non-pressure-related detrimental effects on the kidneys [29], characterized as renal hypertrophy and fibrosis [30,31]. It has been suggested that there are various mechanisms in these adverse effects of dietary salt excess on the kidney. Of those, it has been noticed that the renin-angiotensin system (RAS) is involved in their development. This is because RAS blockade prevents or ameliorates sodium-induced renal damage in salt-loaded SHR without affecting BP [29], suggesting that salt itself is associated with RAS, independent of BP. Here, it is important that the association of salt with renal injury is due to “intra-renal” RAS, but not circulating RAS. Indeed, a basic study has demonstrated that despite a suppression of circulating angiotensin II with salt loading, locally generated “intra-renal” RAS was stimulated by salt and angiotensin II content of proximal tubular fluid increased [32]. Furthermore, Susic et al. [33] demonstrated that RAS activity in SHR was not suppressed or even augmented after four weeks of salt loading, indicating that maintained “intra-renal” RAS combined with a high-salt diet contributes to greater renal damage in SHR.

In addition, oxidative stress is associated with renal damage after salt loading [34]. Lai et al. reported that the increased reactive oxygen species (ROS) plays a role in a high salt-induced renal injury in mice, showing reduced renal mass [35]. Under normal conditions, a low level of oxidative stress is maintained by the balance between the production and the degradation of ROS such as superoxide (O^2−^). That is, reactive O^2−^ is rapidly reduced by the enzyme superoxide dismutase, etc.; however, O^2−^ activity is enhanced when the balance is lost [36,37]. Similar relationship was observed in partial (5/6) nephrectomy in the presence of a high-salt intake model [38] as well as a Dahl salt-sensitive hypertension model [39]. In these models, significant renal damage are accompanied by an increased tissue oxidative stress independent of BP.

## 3. Association of High-Salt Diet with Blood Pressure and Chronic Kidney Disease in Normotensive Individuals and Animals

### 3.1. Association of High-Salt Diet with Blood Pressure in Normotensive Individuals

In normotensive individuals, only a small alteration in BP is observed with the gain or reduction of dietary sodium intake [40]. This phenomenon was confirmed by Parfrey et al. [41] They reported that 28 normotensive individuals undergoing high sodium intake (350 mmol/day) for five days and low sodium intake (10 mmol/day) for five days showed an extremely slight (not significant) fall in mean BP from the high-sodium to the low-sodium diet [41]. Thus, the response to salt intake is largely different between normotensive individuals and hypertensive patients. One reason is due to difference in race. In line with this, Todd et al. [42] reported that participants were all White (European origin), and thus would be expected to be less sodium-sensitive compared with a study that included subjects of African or Asian descent [43].

In general, in response to high salt intake, normal individuals are acutely and chronically resistant to salt-induced hypertension. This is because normal individuals rapidly excrete salt and retain little of it, so that their blood volume does not increase, and therefore BP does not increase. Furthermore, as reported by Crowley and Coffman [44], classic Guytonian models suggest that salt sensitivity is based on a defect in sodium excretion via the kidney. This is, impaired elimination of sodium during high-salt feeding leads directly to expanded extracellular fluid volume, which promotes increased BP. In addition, in the general population, genetic variants associated with decreased activity of sodium transporters in the renal tubule contribute to resistance from hypertension by promoting increased salt excretion and decreased external salt balance [45].

### 3.2. Association of High-Salt Diet with the Kidney in Normotensive Individuals

Todd et al. [42] reported that dietary salt intake did not cause significant elevation in BP in normotensive individuals, but renal parameters were not estimated such as serum creatinine and/or creatinine clearance, so the effects of salt on renal function in normotensive individuals remains unknown.

### 3.3. Association of High-Salt Diet with BP and the Kidney in Experimental Normotensive Model

In normotensive WKY, the various dietary sodium manipulations did not cause any changes in systemic and regional hemodynamics. Similarly, we showed that salt-loaded WKY did not exhibit an elevation of BP, but did display renal histopathological changes, due to the direct effect of salt [46]. In addition, ROS by NADPH oxidase in the blunted renal autoregulatory behavior was observed in Sprague-Dawley rats, a salt-resistant strain of rats, fed high-salt diet [47].

## 4. Biomarkers of Salt-Induced Damages in the Progression to Chronic Kidney Disease

### 4.1. Characteristics of Damage under High Salt Intake and Its Mechanism

Both in SHR and WKY, a high salt intake induced renal tubular injury [48]. Renal tubular damage leads to tubulointerstitial injury and renal fibrosis [49], which is a common pathway for the progression to CKD in spite of renal primary diseases [50]. The tubulointerstitial infiltrate in patients with progressive renal disease consists of monocytes and T lymphocytes [51], and these immune cells are recruited by leukocyte adhesion molecules and chemokines which are expressed in the tubular epithelial cells [52]. In addition, a high salt intake enhances renal injury due to increased oxidative stress in hypertensive [53] rats as well as normotensive [54] rats. It has been reported that the small GTPase Rac 1, a regulatory subunit of NADPH oxidase, is activated by elevated sodium [55], and the Rac1 inhibitor decreased sodium-induced superoxide generation [56]. Until now, it has been widely recognized that a relatively high level of ROS causes redox imbalance, leading to the induction of cell apoptosis or necrosis under various physiological and pathological conditions [57]. Recent reports have shown that ROS can activate mitogen-activated protein kinase (MAPK) and apoptotic cell death induced by ROS is mediated by the p38-MAPK pathway in lung cells [58], and in renal cells [59]. Interestingly, in the kidney of CKD rats, the levels of ROS were markedly elevated, and the activities of anti-oxidative enzymes such as super oxidative dismutase (SOD) and glutathione peroxidase (GSH-Px) showed a significant decrease [59], suggesting that ROS could induce apoptosis.

### 4.2. Biomarkers of Damage under High Salt Intake

In recent years, several novel biomarkers for renal tubular injury have been reported. Among those, urinary kidney injury molecule-1 (Kim-1) [60] and neutrophil gelatinase-associated lipocalin (NGAL) [61] have been investigated. Previously, we found that urinary vanin-1 elevates before the increase of conventional biomarkers for renal tubular damage in rats with nephrotoxicant- and drug-induced renal tubular injury [62,63]. Vanin-1 is an epithelial glycosylphosphatidylinositol (GPI)-anchored pantetheinase [64,65], which catalyzes the conversion of pantetheine into pantothenic acid, vitamin B5, and cysteamine [64,65]. Cysteamine also decreases the protective activities of SOD and GSHPx against ROS toxicity, causing free radical production to overwhelm antioxidant defense systems [66]. Thus, vanin-1 is involved in oxidative stress in tissues [67].

**Figure 1:**
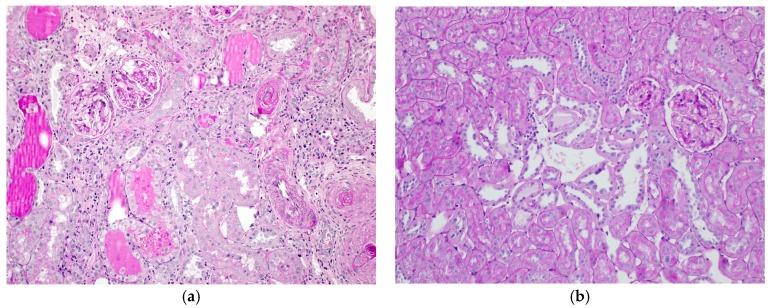
Pathological changes of renal cortical sections in spontaneously hypertensive rats (**a**) and normotensive Wistar Kyoto rats (**b**) after 8%-salt loading for eight weeks. periodic acid-Schiff stain. Original Magnification 200×. Original Image.

Our previous studies [48] revealed the following observations in SHR and/or WKY; (1) a high-salt diet caused severe histopathological renal tubular injury in SHR (Figure 1a), which appeared earlier than the appearance of albuminuria and a decrease in GFR, (2) WKY also exhibited morphologically renal tubular alterations (Figure 1b) under a high-salt diet, which were mild compared to those in SHR, and (3) Urinary excretions of vanin-1 and NGAL elevated after salt-loading both in SHR and WKY. On the other hand, urinary Kim-1 showed a significant increase in salt-loaded SHR after albuminuria, not in salt-loaded WKY. Thus, our studies indicate that urinary vanin-1 and NGAL are earlier biomakers of salt-induced renal tubular damage compared to urinary Kim-1 both in SHR and WKY. That is, urinary vanin-1 and NGAL could be useful for the detection of salt-induced renal injury in both hypertensives and normotensives. In hypertensive patients, salt-intake elevates BP, which is a sign of renal injury induced by salt, whereas normotensive individuals are unlikely to notice such injuries as they exhibit no change in BP. In light of the clinical difficulties to cure massive kidney injury, markers for the prediction of kidney injury, detected in pre- or early stages of kidney injury, may be more important than regular biomarkers. Vanin-1 could be serve as a predictor of kidney damage.

## 5. Conclusions

Urinary vanin-1 and/or NGAL could be useful biomarkers for detecting renal tubular injury caused by high salt intake at an early stage, before the progression to CKD, both in hypertensive and normotensive animals. Clinical studies will be needed to clarify whether these biomarkers will be useful for the detection before the progression to CKD.

## Abbreviations

BP	Blood pressure
CKD	Chronic kidney disease
Kim-1	Kidney injury molecule-1
NAG	*N*-Acetyl-β-d-glucosaminidase
NGAL	Neutrophil gelatinase-associated lipocalin
RAS	Renin-angiotensin system
ROS	Reactive oxidative species
SHR	Spontaneously hypertensive rats
WKY	Wistar kyoto rats
